# MUC16 expression in Sjogren’s syndrome, KCS, and control subjects

**Published:** 2008-12-30

**Authors:** Barbara Caffery, Elizabeth Joyce, Miriam L. Heynen, Lyndon Jones, Robert Ritter, Daniel A. Gamache, Michelle Senchyna

**Affiliations:** 1 Center for Contact Lens Research, School of Optometry, University of Waterloo, Ontario, Canada; 2 Alcon Research Ltd, Fort Worth, TX

## Abstract

**Purpose:**

To investigate the expression of MUC16 protein in tears and conjunctival cell membranes and *MUC16* mRNA in conjunctival cells of Sjogren’s syndrome (SS), keratoconjunctivitus sicca (KCS) and non-dry eyed (NDE) subjects. The relationship of tear flow and soluble MUC16 concentration was also measured.

**Methods:**

Seventy-six subjects were recruited for this study: 25 SS (confirmed via American-European Consensus Criteria 2002), 25 KCS (confirmed by symptoms and Schirmer scores ≤10 mm) and 26 NDE. Tear flow was measured by the Schirmer test without anesthesia for 5 min. Tears were collected using an eye-wash technique. Protein and mRNA were isolated from conjunctival epithelial cells collected via impression cytology. Soluble and membrane bound MUC16 were quantified via western blotting and *MUC16* mRNA was quantified by real time qPCR.

**Results:**

The SS group demonstrated significantly higher concentrations of soluble MUC16 (7.28 [SS]±3.97 versus 3.35 [KCS]±4.54 [p=0.004] and versus 1.61 [NDE]±1.22 [p<0.001]) and *MUC16* mRNA (4.66 [SS]±5.06 versus 1.84 [KCS]±2.26 [p=0.01] and 1.52 [NDE]±1.04 [p=0.003]) compared to both KCS and NDE groups, respectively. No differences in soluble MUC16 or *MUC16* mRNA were found between the KCS and NDE groups. Membrane bound MUC16 was similar in all three groups. No significant correlation was found between mean Schirmer values and any measure of MUC16 expression.

**Conclusions:**

These results demonstrate that SS subjects display a significant increase in both soluble MUC16 and *MUC16* mRNA concentrations compared to other forms of aqueous deficient dry eye and non dry-eyed individuals. There was no correlation between tear flow and soluble MUC16 concentration.

## Introduction

Mucins constitute an important part of the preocular tear film and ocular surface. Our current understanding is that the tear film is best described as a mucin/aqueous gel decreasing in density toward the lipid layer [1,2]. Mucins are believed to play a key role in the retention of water and other tear fluid components on the ocular surface, hence maintaining both lubricity of the ocular surface and a healthy epithelial barrier [3,4]. Early concepts of ocular mucins described goblet cells as the sole origin of secretion [5]. However, numerous studies have now shown multiple species of mucins are also derived from the ocular surface epithelium [6-10].

To date, 20 human mucin genes have been completely or partially sequenced. They have been named and numbered chronologically with their discovery: *MUC1*, *MUC2*, *MUC3A*, *MUC3B*, *MUC4*, *MUC5AC*, *MUC5B*, *MUC6–9*, *MUC11–13*, *MUC15–17*, and *MUC20* [11-19].

Based on the presence of common structures within their amino acid sequences, they have been grouped together into three broad groups [6,11,14-20]:

1. Gel-forming mucins from goblet cells of various epithelia: MUC2, MUC5AC, MUC5B, MUC6, and MUC19
2. Soluble mucins: MUC7 and MUC9
3. Membrane-associated mucins: MUC1, MUC3A, MUC3B, MUC4, MUC11, MUC12, MUC13, MUC15, MUC16, MUC17, and MUC20.

Both secreted (MUC2, MUC5AC, MUC5B, and MUC7) and membrane bound (MUC1, MUC4, and MUC16) mucin forms have been reported to be expressed by ocular surface epithelia [6,21,22]. Of the mucins identified on the ocular surface, two soluble (MUC2 and MUC5AC) and three membrane-bound (MUC1, MUC4, and MUC16) forms are considered “critical” for the maintenance of a “normal” tear film [21]. MUC2 and MUC5B are present in very low quantities [21-23].

Recent research has demonstrated that alternative soluble forms [23-27] of MUC1 [24] and MUC16 exist, as has been illustrated by Spurr-Michaud et al. [23],  who described soluble MUC16 in the tears. The alternative forms of MUC1 and MUC16 lack the cytoplasmic tail portion of the protein and thus are secreted or shed into the tear film, as opposed to being anchored on epithelial cell membranes. Whether these soluble species are present in all tear samples or are linked to ocular surface pathology is not yet clear. Also unclear is the functions attributed to these alternative, soluble forms of MUC1 and MUC16.

The functions of MUC16 are slowly becoming understood. Blalock et al. [22] using immortalized corneal-limbal epithelial cells (HCLE), suggested that MUC16 forms a protective barrier on these cells, without which there is rose bengal dye penetrance and adherence of *S. aureus*. They locate MUC 16 on the tips of the HCLE cell surface and suggested that the cytoplasmic tail binds to the actin cytoskeleton. No such work has been done with human conjunctival cells.

Although alterations in mucin expression and/or mucin glycosylation have been implicated in the pathophysiology of dry eye, only a limited number of studies addressing these issues have been conducted. The recent DEWS report eliminated the separate classification of primary mucin deficient dry eye [28], with their classification supporting both aqueous-deficient (AD) and evaporative (E) dry eye groups. Within the AD dry eye group, two major subclasses exist, namely Sjogren’s Syndrome (SS) dry eye and Non-SS dry eye.

The role of mucins in dry eye disease has mainly focused on MUC5AC, and it is generally assumed that the expression of MUC5AC is reduced in dry eyed subjects [29]. Recently, attention has also been placed on membrane spanning mucins and their potential role in dry eye [22,30]. Danjo et al. [30],  have reported that the conjunctival epithelial cell distribution of H185 (MUC16) is altered in non-SS dry eye subjects.

It is the membrane spanning mucin MUC16 that is the subject of this paper. Specifically, we sought to characterize the expression of MUC16 in Sjogren’s syndrome dry eye as compared with aqueous deficient (KCS) dry eye and non dry-eyed (NDE) controls, to gain further insight into the role that MUC16 may play in dry eye disease. In addition, we examined the relationship between soluble MUC16 expression and tear flow as measured by Schirmer testing.

## Methods

### Study Design

Prior to the start of this study, ethics approval was attained from the Office of Research Ethics at the University of Waterloo and University of Toronto and all procedures adhered to the Declaration of Helsinki. A total of 76 subjects (26 non dry-eyed controls [NDE], 25 Sjogrens subjects [SS], and 25 non-Sjogrens keratoconjunctivitis [KCS]) were enrolled in this study. All participants underwent a clinical evaluation visit to determine entry eligibility before a second visit in which ocular samples were collected.

All SS participants had been diagnosed with primary SS at the Sjogren’s Syndrome Clinic of the Toronto Western Hospital, using the American-European consensus criteria of 2002 [31]. Thus, each of these subjects had 3 or more of the following criteria: symptoms and signs of dry eye and dry mouth and each had one of: a positive minor salivary gland biopsy or the presence of SS specific antibodies Ro and/or La in the serum. Recruitment of these patients was achieved through telephone calls from the database of that clinic. No further preliminary screening was performed on this group as all had confirmed Sjogren’s Syndrome.

The KCS and NDE subjects were recruited through the SS clinic and a private practice. Participants first answered the question “If you have dry eyes, have they been dry for at least 3 months”? Participants that answered “yes,” were asked to rate their dryness on a visual analog scale used routinely in the SS clinic. The horizontal line of the scale was marked from 0 to 10. At the 0 point the words “not dry at all” were written and at the 10 “as dry as the desert” was written. If they rated their dryness as greater than or equal to 6 out of 10 on the visual analog scale and the Schirmer 1 test score was less than or equal to 10 mm in 5 min in at least one eye, they were classified as KCS. Non-dry eye subjects (NDE) were enrolled if they stated that they did not have dry eyes, ranked their dryness as 0 on the visual analog scale and had Schirmer I scores of greater than 10 mm in both eyes. All subjects were free from allergy or other ocular surface diseases and all were on maximum therapy for blepharitis, if that condition had been previously diagnosed. Thus, where appropriate, they were using lid scrubs and hot soaks but were not using topical antibiotics or topical anti-inflammatories. Pre-screening for KCS and NDE subjects was performed within two months of the actual clinic visit for collection of tears and impression cytology specimens. Participants were required to confirm that their dry eye status had not changed at the collection visit to be included in the study.

### Reagents and Materials

Agarose was purchased from Cambrex Bio Science (Rockland, ME). Gel buffer, tank buffer, vacuum blotter, nitrocellulose membrane and, blotting paper were purchased from BioRad (Mississauga, ON, Canada). Glycerol and 20X SSC transfer buffer were purchased from VWR (Mississauga, On, Canada). Molecular weight standards (Himark™) were purchased from Invitrogen (Carlsbad, CA). ECL-Plus™ lits were purchased from GE Healthcare (Baie d”Urfe, QC, Canada). DC Protein Assay Kits® were purchased from BioRad. Monoclonal mouse anti-human MUC16 antibody (OC125) was purchased from DAKO (Glostrup, Denmark) and goat anti-mouse IgG-HRP from Santa Cruz Biotechnology Inc. (Santa Cruz, CA). Millipore™ Membrane Filters were purchased from Millipore™ (Billerica, MA)

### The Schirmer I test

The Schirmer I test was performed using pre-packaged, sterile paper strips (Schirmer Tear Test Strips®; Alcon, Fort Worth, TX), without anesthesia. The rounded bulb end of the strip was folded at the notch and then inserted into the lower fornix, one third of the distance from the lateral canthus of the lower lid. Once both strips were in place, the subject was asked to close their eyes. After 5 min the strips were removed and the wet portion measured in mm.

### Eye wash tear collection

Tears were collected using an eye wash method as described elsewhere [29]. Briefly, 60 µl of sterile, physiologic saline (0.9% NaCl; Minims; Chauvin Pharmaceuticals Ltd, Romford, Essex, UK) was applied to the superior bulbar region of the unanaesthetized ocular surface (right eye) using a sterile micropipette. Participants were asked to rotate their eyes without blinking (lids were still held open), to mix the tear fluid. Tear washes were collected from the inferior fornix of each eye using the sterile micropipette. The same procedure was repeated with the left eye. Both eye washes were pooled together, vortexed briefly, then placed on dry ice until transfer to −80 °C for storage.

### Conjunctival impression cytology (CIC)

Conjunctival epithelial cells were collected via impression cytology from each eye using sterile Millipore, MF membranes, (pore size 0.45 μM). Two drops of a topical anesthetic (Alcaine®, Alcon), dosed 60 s apart, were applied to the right eye. Fifteen seconds after the second drop of anesthetic, the subject was instructed to hold gaze down to expose the superior conjunctiva. The investigator held the upper lid up to fully expose the superior conjunctiva. One piece of filter paper was placed on the superior region of the conjunctiva for five to seven seconds then removed with blunt forceps and placed in a sterile pre-labeled 2 ml capped polypropylene centrifuge tube containing 1 ml of RLT® RNA Isolation Buffer (Qiagen, Germantown, MD) containing 0.01% β-mercaptoethanol. The same procedure of impression cytology then took place on the temporal conjunctiva and the filter paper was placed in the same tube as the superior sample. Anaesthesia and impression cytology of the left eye then took place as described for the right eye, with the exception that the two filter papers were placed in an empty sterile 2 ml capped polypropylene centrifuge tube, such that protein extraction could take place. All samples were immediately placed on dry ice, then transferred to −80 °C for storage until processing.

### Protein isolation from CIC samples

Impression cytology filter papers that were collected from the left eyes of subjects were used to isolate total protein. Filter papers were placed cell side up on small glass plates and 5 µl of extraction buffer (2% SDS; 1X Complete™ protease inhibitor cocktail [Roche, Mannheim, Germany]) was placed on each. Using a steel scalpel blade, each membrane was cut into small pieces, which were placed in 600 μl capped polypropylene centrifuge tubes and covered with an additional 50 µl of extraction buffer. Tubes were vortexed, then heated at 95 °C in a heating block for 10 min. Tubes were centrifuged at 12,000x g for 6 min to pellet the filter pieces and the protein extract was collect and transferred to a fresh capped polypropylene centrifuge tube. Extraction buffer (20 μl) was added to the pelleted filter paper. Following vortex and centrifugation, wash was collected and added to the first protein aliquot.

### Determination of total protein concentration in tear and CIC samples

All total protein determinations were conducted using the DC Protein Assay Kit® (BioRad), following manufacture’s instructions. For samples that contained SDS, 20 µl of Reagent S was added to each ml of Reagent A. Five µl each of eye wash or impression cytology extract was added to 5 µl of Milli-Q water and the final 10 µl was divided equally between two microplate wells to allow assay in duplicate. Absorbances were read at 750 nm on a Multiskan Microplate Spectrophotometer (Thermo Fisher Scientific, Mississauga, Ontario, Canada).

### Electrophoresis and immunoblotting

Samples were thawed at room temperature and diluted 4:1 with 5X sample buffer (247 mM Tris-HCl, pH 8.6, 2% SDS [w/v], 50 mM DTT, 1X Complete™ Protease Inhibitor [Roche, Mannheim, Germany], 10% glycerol, 0.002% [w/v] Bromophenol blue). Samples were further diluted with 1X sample buffer to achieve a final protein concentration of 1 μg/μl. Samples were heated at 100 °C for 3 min, cooled to room temperature than placed on ice. MUC16 standard antigen (CA125; 1–60 units/well) was run on each gel to normalize data and facilitate semi-quantitation of samples, through linear regression analysis. Ten µg/lane of eye wash protein and 5 µg/lane of CIC total protein was loaded per lane. Following separation, protein was transferred to nitrocellulose membranes via vacuum transfer with 4X SSC buffer for 2 h. Membranes were fixed by heating at 70 °C for 30 min. Membranes were air dried for 12 h then blocked in PBS + 0.05% Tween-20 (=PBS-T) + 0.1% BSA + 10% NAP (NAP-Blocker; G Biosciences, Maryland Heights, MD), for 1 h at room temperature on an orbital shaker. Following three washes with PBS-T, blots were incubated overnight in mouse monoclonal antibody clone OC125, (1: 250) in PBS-T and 0.1% BSA + 10% NAP at 4 °C. After rinsing in PBS-T, blots were incubated with the secondary antibody (1:5,000) in PBS-T + 0.1% BSA + 10% NAP for 1 h at room temperature. Blots were developed with ECL® (BioRad) and chemiluminescent signals were captured by Storm840® Imaging (Molecular Dynamics, Sunnyvale CA). The amount of MUC16 in each sample and standard were quantified by image analysis software (ImageQuant 5.1®; Molecular Dynamics). Known amounts of CA125 standard were used to generate standard curves and using the line-of-best-fit from the standard curve, the relative amount of mucin in the samples was interpolated from the graph. It should be noted that samples often produced multiple chemiluminescent signals of varying molecular weights. For quantitation, only signals above 300 kDa for MUC16 were used.

### RNA isolation from CIC samples and reverse transcription

Tubes containing 1 ml of RLT® buffer (Qiagen) and two impression cytology samples collected as described above were allowed to thaw at room temperature then vortexed for 30 s. Membranes were removed using a 21 guage needle and samples were vortexed again and then passed through a 21 gauge needle for 10 times. Extraction of total RNA proceeded according to manufacturer’s directions (RNeasy® Minikit; Qiagen). The DNase step, as recommended, was performed. The final isolation step was conducted with 40 µl of RNase free water. Following a 1 min centrifuge step (8,000x g), flow-through (total RNA) was collected and stored at −80 °C.

RNA quantity and quality were assessed by measuring the optical density using a Beckman DU530 Life Science UV/Visible Spectrophotometer at 260 nm and 280 nm. DNA was synthesized from 8 µl of RNA sample using random hexamer primers with Superscript™ III First-Strand Synthesis System for RT–PCR (Invitrogen, Carlsbad, CA) according to the manufacturer’s instruction.

### Real time-qPCR

Relative expression of genes of interest was performed in multiplex PCR reactions containing target (*MUC16*) and endogenous control (*GAPD*H) oligonucleotide primers in the presence of gene-specific dye-labeled Taqman probes (Table 1). Two microliters of cDNA was used for amplification in a 50 µl PCR reaction containing target (300 nM) and endogenous control (100 nM) oligonucleotide primers, control and target Taqman probes (100 nM), and Taqman^®^ Universal PCR Master Mix (Applied Biosystems, Foster City, CA). Duplicate samples were used for analysis in a 7500 Real Time PCR System (Applied Biosystems). Conditions used for amplification were as follows: 50 °C for 2 min, followed by an initial 10 min denaturing step at 95 °C. This was followed by 40 cycles of denaturing at 95 °C for 30 s, annealing at 60 °C for 30 s, and extension at 72 °C for 45 s. Normalized reporter dye fluorescence (R_n_) data was collected during the extension step at each cycle.

**Table 1:** Sequence data for gene amplification in real time RT–PCR.

**Gene**	**Forward primer**	**Reverse primer**	**Taqman probe**
*MUC16*	ACCCAGCTGCAGAACTTCA	GGTAGTAGCCTGGGCACTGT	6FAM-GCGGAAGAAGGAAGGAGAAT
*GAPDH*	GAAGGTGAAGGTCGGAGTCA	GACAAGCTTCCCGTTCTGAG	VIC-CAATGACCCCTTCATTGACC

Collected data was analyzed and fold-expression changes were calculated using the comparative method (2^-ΔΔCT^) of relative quantification by SDS software (v1.3.1; Applied Biosystems). A sample containing 0.25 pg of plasmid DNA with cloned target and endogenous fragments was used as a calibrator sample for each gene.

### Data analysis

Statistical analysis was performed using Statistica Ver7.1 (StatSoft Inc., Tulsa, OK) and Microsoft Excel™ XLfit© software. Graphs were plotted using Statistica Ver7.1. All data are reported as mean±standard deviation. Statistical differences between groups for biomarker data were identified by using one-way ANOVA, and when necessary, Dunnett’s comparison of means and by Tukey’s test. Significance was identified at p<0.05 (α=0.05).

## Results

### Demographics and tear flow measurements

A total of 76 subjects were enrolled into this study and the subject demographics are displayed in Table 2. The mean age of the SS group was found to be statistically higher than the NDE group (p=0.024), but not different from the KCS group (p>0.05). Mean Schirmer I scores from both eyes collected without anesthesia for five min revealed a significantly reduced (p<0.0001) tear flow in both SS (5.12±5.96 mm) and KCS subjects (7.84±7.35 mm), relative to NDE (23.83±7.85 mm). There was no difference in mean Schirmer I scores between the KCS and SS groups (p=0.19).

**Table 2:** Summary of demographic information for study groups.

**Group**	**Mean age (years)**	**Number of female subjects**	**Number of male subjects**	**Total subjects**
NDE	52.4±11.4	24	2	26
KCS	59.3±9.1	21	4	25
Sjogren’s syndrome	60±11.8*	21	4	25

The asterisk denotes significantly greater compared to control group (p=0.024).

### MUC16 results

**Figure 1:**
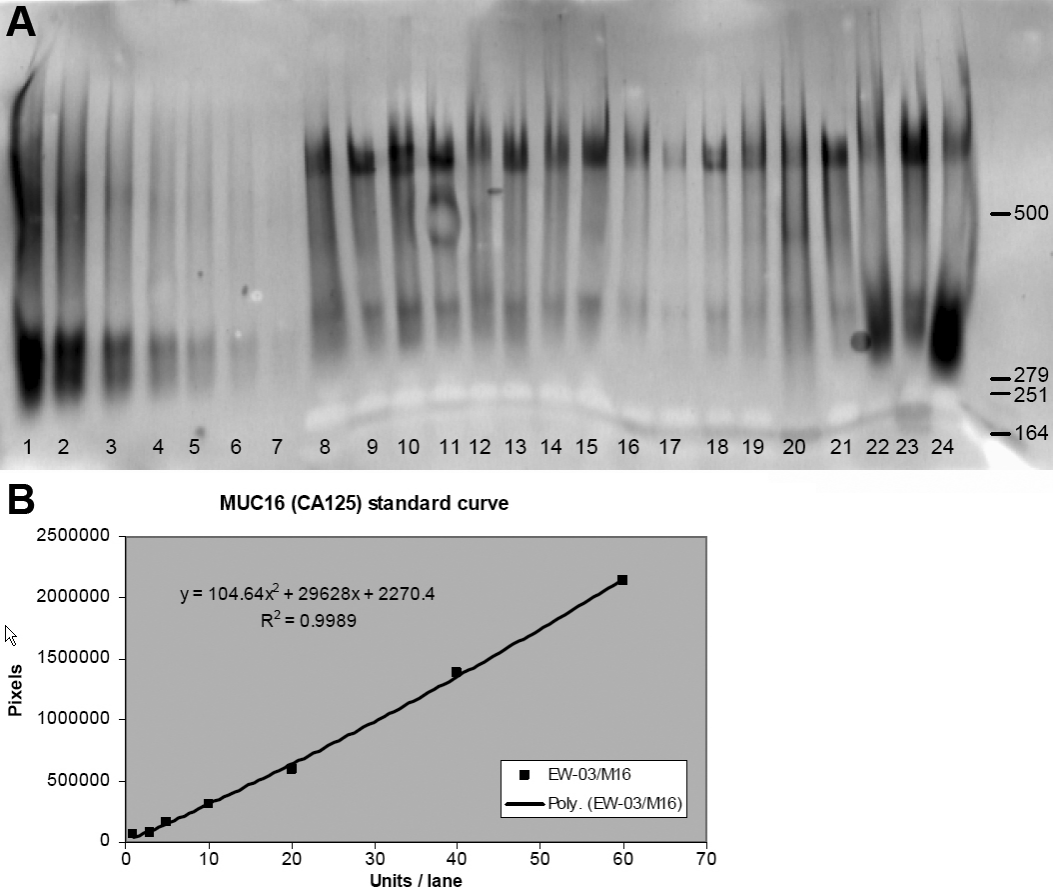
Western blot and regression analysis for soluble MUC16 quantification. **A**: An example of a soluble MUC16 western blot from tear samples derived from 17 subjects. Lanes 1–7 are MUC16 standard antigen  (CA125) Units (based on radio-immunoassay calibration from the vendor); (Lane 1=60, Lane 2=40, Lane 3=20, Lane 4=10, Lane 5=5, Lane 6=3, Lane 7=1 U); Lanes 8 - 24 are tear samples. **B**: A regression curve was created by graphing applied concentration of CA125 standard against the optical density of the resulting band immunoreactivity. Total MUC16 concentration was quantified by extrapolation from this curve using all signal above 300 kDa.

**Figure 2:**
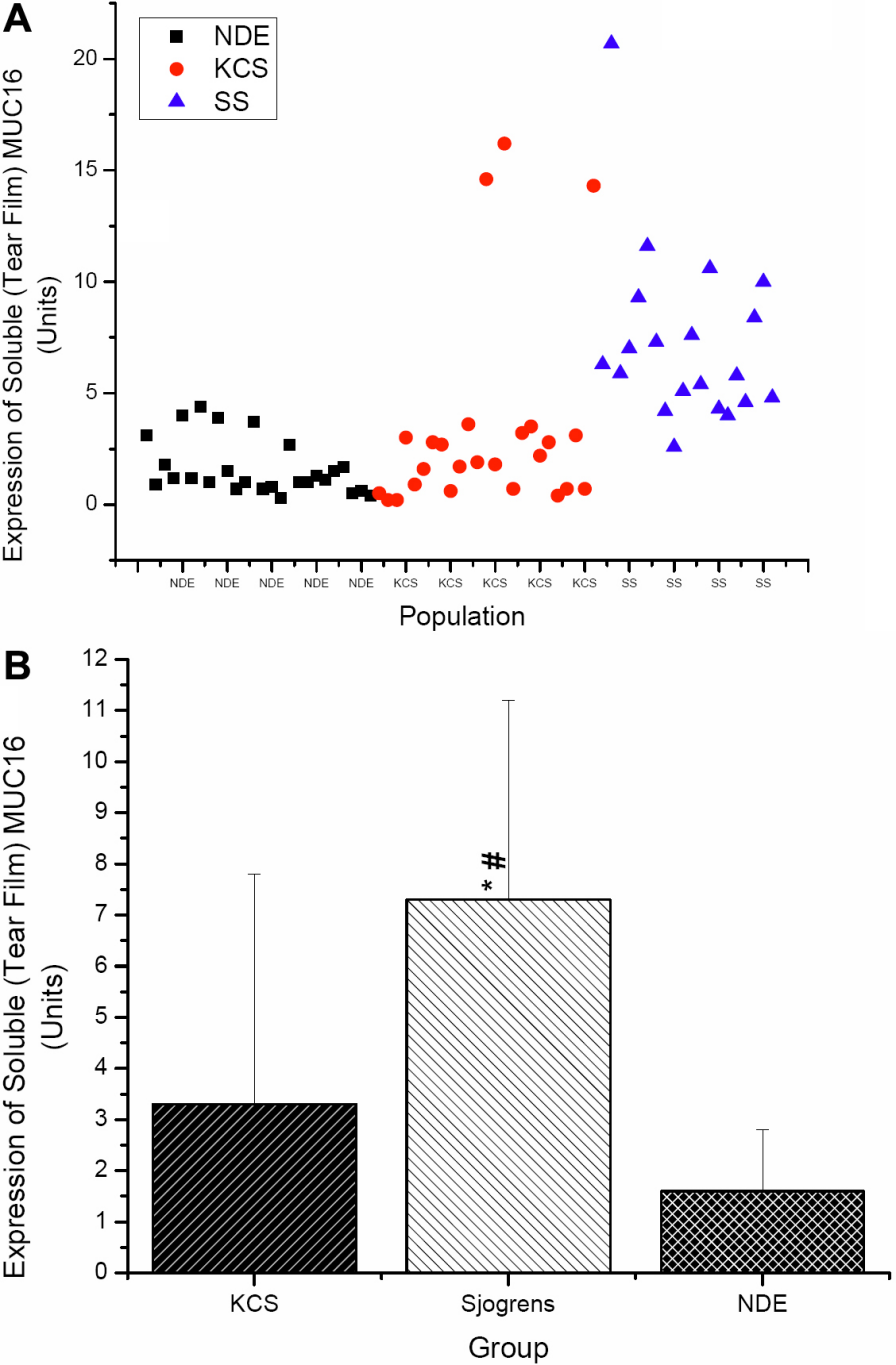
Soluble MUC16 expression as quantified by western blotting. Data expressed as scatter graph of individual data points (**A**) and mean data (**B**). Protein samples collected via eye wash and MUC16 data expressed in Units as calculated from extrapolation from a standard curve titration of CA125. The asterisk indicates significantly different compared to the NDE group while the sharp (hash mark) indicates significantly different compared to the KCS group.

**Figure 3:**
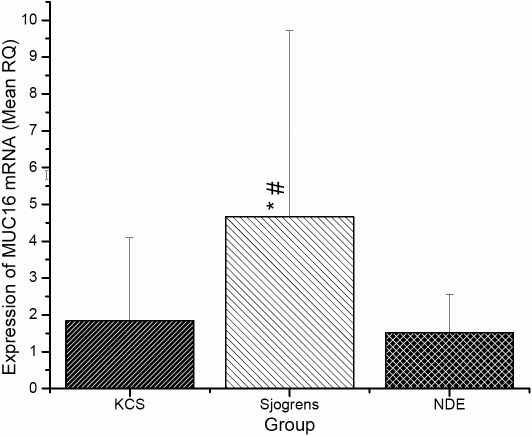
Summary of *MUC16* mRNA expression as quantified by qPCR. RNA isolated from conjunctival epithelial cells collected via impression cytology. The asterisk indicates significantly different compared to the NDE group while the sharp (hash mark) indicates significantly different compared to the KCS group.

**Figure 4:**
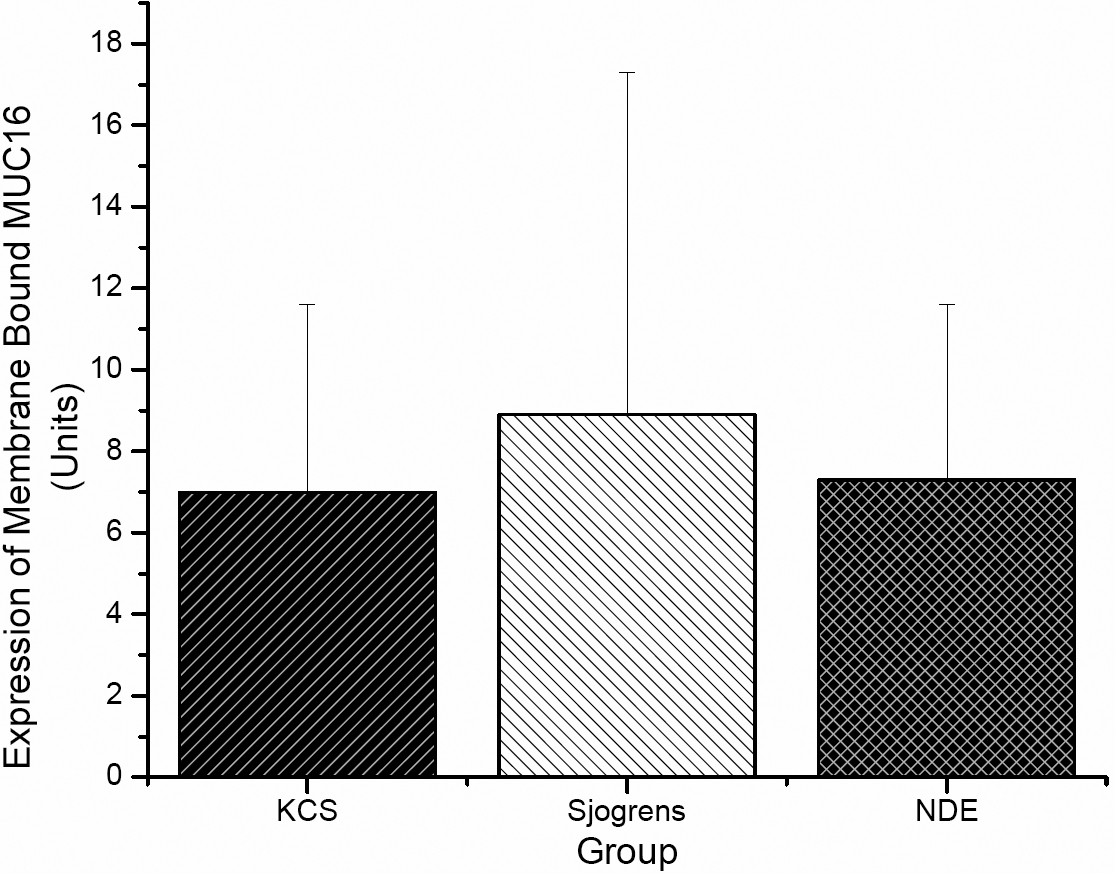
Summary of membrane bound MUC16 as quantified by western blotting. Protein samples collected via impression cytology. MUC16 data expressed in Units as calculated from extrapolation from a standard curve made from titration of CA125.

Five of the 25 SS subjects did not supply sufficient tear samples for analysis of soluble MUC16, limiting this analysis to 20 of the 25 subjects. Representative data are displayed in Figure 1. All samples that were analyzed displayed quantifiable amounts of soluble MUC16, although significant inter-sample variation was observed both in terms of migration pattern of the MUC16 signal on western blots (Figure 1A) and total amount of MUC16 (Figure 2A). Mean data showed that the SS group demonstrated significantly higher concentrations of soluble MUC16 compared to both KCS (7.28±3.97 versus 3.35±4.54; p=0.004) and NDE (7.28±3.97 versus 1.61±1.22; p<0.0001) groups (Figure 2B). The SS group also demonstrated a significantly higher concentration of *MUC16* mRNA compared to both KCS (4.66±5.06 versus 1.84±2.26; p=0.01) and NDE (4.66±5.06 versus 1.52±1.04; p=0.003) groups (Figure 3). No differences in the concentrations of soluble MUC16 and *MUC16* mRNA were found between the KCS and NDE groups (p>0.05). A weak (r2=0.13), but significant correlation was found between the expression of soluble MUC16 protein and mRNA. Lastly, no difference between membrane bound MUC16 was found between any groups (p>0.05; Figure 4).

A single significant correlation was found between mean Schirmer values compared with any measure of MUC16 expression; that being soluble MUC16 concentration in the combined KCS and NDE groups (Table 3).

**Table 3:** Slope and correlation data summary for comparison of MUC16 expression data with mean Schirmer scores.

**Subject group**	**Soluble MUC16 versus** **Schirmer score**	**Membrane bound MUC16 versus Schirmer score**	***MUC16* mRNA versus** **Schirmer score**
Sjogren’s data	y=0.02x + 7.15 r2=0.001; p=0.87	y=0.22x + 7.73 r2=0.02; p=0.46	y=0.05x + 4.47 r2=0.004 p=0.77
KCS and NDE data	y=-0.1x + 4.04 r2=0.09; p=0.03	y=-0.04x + 7.9 r2=0.01; p=0.47	y=0.004x + 1.6 r2=0.0005; p=0.88

## Discussion

The results of this study demonstrate that subjects with confirmed Sjogren’s Syndrome display a significant increase in MUC16 expression, as the concentrations of both MUC16 soluble protein and mRNA were found to be higher than those of aqueous deficient dry eye subjects (KCS) and subjects with no dry eye disease (NDE). There were no differences found in membrane bound MUC16 protein concentration between any of the groups and no difference in any form of MUC16 found between the KCS and NDE groups. Lastly, no correlation between tear flow and MUC16 expression was found in any group.

The numbers of subjects in this pilot study were small. Not all SS subjects (5/25) were able to provide sufficient material for full biochemical analysis. Specifically, total tear protein collected in the eye wash was insufficient, either due to very low protein concentration of the tears or low recovery volume. It is our experience that on very dry eyes, a large fraction of the applied saline is absorbed by the ocular surface. Thus, given the potential relevance of our findings, further investigation of these MUC16 in larger groups is warranted.

We note that age may have been a factor in our results. The mean age of the two dry-eyed populations was greater compared to the control group, with significance reached in the SS group. We cannot totally rule out that this age difference may have influenced our findings, as the literature does suggest that tear volume, production stability and/or quality is reduced in the older population [32,33]. It is not known how mucin changes with age on the ocular surface. However, in our study, there was no significant age difference between the KCS and SS groups that did show differences.

This group of primary Sjogren’s Syndrome patients was well defined, as the diagnosis was confirmed by the American-European consensus criterion of 2002 [31]. Our KCS group had Schirmer test confirmed reduced aqueous secretions and thus we were able to compare the two well defined aqueous deficient groups as stated in the DEWS 2007 definition of dry eye [28].

Others have explored transmembrane mucin expression in SS and dry eye disease. Data reported by Spurr-Michaud et al. [23] describing the presence of MUC16 in the tear film is confirmed by our results. That MUC16 is one of the trans-membrane mucins of the surface conjunctival epithelium was determined by Argueso et al. [9] and its presence within the conjunctival epithelium in both protein and mRNA is confirmed with this study. There is, however, very little additional work on MUC16 in SS. Data on other membrane bound mucins has suggested that the expression of mucosal epithelial membrane mucin (as detected by an uncharacterized antibody referred to as AMEM2), is reduced in SS and non-SS dry eyed subjects compared to controls [34]. At the genetic level, a specific splice-variant of *MUC1* may be reduced in dry eyed subjects [35], although others authors have failed to find differences in *MUC1* or *MUC4* expression between controls and SS subjects [29].

Our finding of excess MUC16 on the SS ocular surface, was particularly interesting to the clinical author (B.C.) who has long observed that excess ocular mucus is a common clinical finding in SS patients. The sole reference to excess mucus in SS that we have noted is that of “mucous aggregates” adherent to the conjunctival and corneal epithelia in a paper by Pflugfelder et al. [36] in 1990. To our knowledge, this is the first report of an increase in MUC16 in the tear film of SS subjects. The authors also understand that the literature does not characterize SS as a disease of excess mucous production. In fact, most other papers have suggested that dry eye disease is a disease of reduced mucous production and secretion [34,37,38]. Certainly, the secreted mucin MUC5AC has been shown to be reduced in SS tears and conjunctival cells [29].

Alterations in the distribution of conjunctival epithelial MUC16 has been noted in dry eye disease. Danjo et al. [30], using immunolocalization, noted that superficial temporal conjunctival epithelial cells did not bind the H185 antibody (MUC16) in non-SS dry eye as well as in normal subjects. Our western blot analysis failed to support these findings. The H185 epitope has been established as MUC16, however, we cannot rule out that different detection probes may have influenced these results and that the identification of MUC16 may be influenced by the level of glycosylation. That differential glycosylation of MUC16 may be a relevant area of study is demonstrated by the significant heterogeneity of signal migration evidenced in western blots. Whether differential glycosylation affects antibody recognition and/or the function properties of the MUC16 protein is not known currently.

The selection of dry-eyed subjects is an important variable in comparing these studies. In part I of Danjo’s [30] study, when differences between dry eyes and normals were studied, subjects were chosen by dry eye symptoms and the presence of rose bengal or fluorescein staining of the ocular surface, in the absence of autoimmune disease. As Schirmer testing was not part of this group’s inclusion criteria, true aqueous deficiency was not established. KCS subjects in the present study were enrolled based on the presence of at least moderate dry eye symptoms and a Schirmer score of less than or equal to 10 mm. Another notable difference is that staining was not an inclusion criterion in our study. Such differences in inclusion criteria make comparisons between published studies somewhat difficult.

Which epithelial cells were used for characterization is also a relevant factor. In this study, impression cytology was performed on both temporal and superior bulbar conjunctival areas and the samples were pooled. Danjo et al. [30], in part I of their study, used cells from the temporal conjunctiva in the dry eye group and a combination of inferior and temporal conjunctiva from the normal controls. Clinically, staining patterns of the conjunctiva are quite different and, as such, it is likely that differential expression of various mucin species may be a function of which cells were analyzed. Comparing mucin expression in exposed and non exposed conjunctival cells would be an important contribution to the literature.

Alterations in membrane MUC16 has been studied in relationship to conjunctival staining. Dry eye patients, particularly those with Sjogren’s syndrome, present clinically with rose bengal staining of the conjunctiva and cornea [31]. Initially rose bengal was thought to stain cells that were desquamated or dead [39-42]. More recently, the dysfunction of mucins has been implicated. Danjo et al. [30] studied aqueous deficient but not SS dry eyed subjects, who had Schirmer scores of less than or equal to 5 mm in 5 min and staining by rose bengal or fluorescein, in part II of their study . They found a significant correlation between staining scores of the temporal conjunctiva and an altered H185 (MUC16) binding pattern. Two studies used a human corneal-limbal epithelial cell line (HCLE) to demonstrate that MUC16 surface protein protects against rose bengal invasion [22,26]. Others have reported a positive correlation between decreased transmembrane mucin (not identified) and higher rose bengal staining in aqueous deficient dry eye [34]. Although we did not stain the ocular surface of the subjects at the time of their visit for tear and cell collection in this study, our results suggest that staining scores, at least in SS patients, would not correlate with a reduction in membrane MUC16, as we found no differences in membrane bound MUC16 expression in any of our subject groups.

The observation of excess ocular mucus in SS patients and the results of this study that show excess MUC16 in the tear film of SS subjects, allowed us to speculate on the possible mechanisms of such mucin production. It appears that there is an active upregulation of mucin production in our SS subjects, as determined by increased mRNA, followed by excess shedding of this mucin into the tear film. Mucin production in humans is an ancient defense mechanism [43] and non-ocular mucous membranes, such as those of the airways, demonstrate excess mucous production under adverse conditions in dogs, rats, and humans [44-46]. The ocular surface performs compensatory mucin related activities in other autoimmune states such as ocular cicatricial pemphigoid (OCP). As the OCP ocular surface moves toward keratinisation, there is increased expression of the family of glycosyltransferases that act at the initial stages of mucin glycosylation [47]. That these findings were found in the early stages of the keratinization process suggests that ocular surface cells can participate in compensatory attempts to synthesize more mucin to maintain a wet surface phenotype. Since our results demonstrated no correlation between tear flow and MUC16 concentration, we believe that it is the unique nature of autoimmune related dry eye that influences the stimulus for MUC16 expression. Perhaps this is a result of a signaling mechanism peculiar to SS, that functions to maintain a more “healthy” ocular surface in the absence of aqueous tears.

Another factor that could increase the concentration of MUC16 on the SS ocular surface is the change in flushing and clearing mechanisms that occur with extreme dryness. Berry et al. [48] suggest that in dry eye, tear mucins may form an irreversible complex with other surface components, which prevents normal removal.

In summary, we quantified the expression of ocular surface MUC16 in Sjogren’s subjects and compared them with non-Sjogren’s dry-eyed subjects and non-dry eyed controls. We found that Sjogren’s subjects express significantly elevated concentrations of both soluble MUC16 and *MUC16* mRNA compared to both KCS and NDE groups. No differences were found in MUC16 expression between the KCS and NDE subjects. All three groups had similar concentrations of membrane bound MUC16. No correlation was found between tear flow and MUC16 expression. We propose that conjunctival cells in Sjogren’s syndrome increase their production of MUC16 as a compensatory mechanism to maintain their healthy phenotype.
